# Effect of acute fentanyl treatment on synaptic plasticity in the hippocampal CA1 region in rats

**DOI:** 10.3389/fphar.2015.00251

**Published:** 2015-10-30

**Authors:** Hai Tian, Yueming Xu, Fucun Liu, Guowei Wang, Sanjue Hu

**Affiliations:** ^1^Clinic of Anesthesiology, No. 324 Hospital of the People's Liberation ArmyChongqing, China; ^2^Clinic of Pharmacology, No. 324 Hospital of the People's Liberation ArmyChongqing, China; ^3^Department of Medical Affairs, No. 324 Hospital of the People's Liberation ArmyChongqing, China; ^4^Institute of Neurosciences, The Fourth Military Medical UniversityXi'an, China

**Keywords:** fentanyl, hippocampus, LTP, fEPSP, μ-opioid receptor, interneuron

## Abstract

Postoperative cognitive dysfunction (POCD), mainly characterized by short-term decline of learning and memory, occurs after operations under anesthesia. However, the underlying mechanisms are poorly understood. The μ-opioid receptors (MOR) are highly expressed in interneurons of hippocampus, and is believed to be critical for the dysfunction of synaptic plasticity between hippocampal neurons. Therefore, we investigated the effect of fentanyl, a strong agonist of MOR and often used for anesthesia and analgesia in clinical settings, on hippocampal synaptic plasticity in the Schaffer-collateral CA1 pathway during acute exposure and washout *in vitro*. Our results revealed that acute fentanyl exposure (0.01, 0.1, 1 μM) dose-dependently increased the field excitatory postsynaptic potentials (fEPSPs), which was prevented by pre-administration of picrotoxin (50 μM) or MOR antagonist D-Phe-Cys-Tyr-D-Trp-Orn-Thr-Phe-Thr-NH2 (CTOP, 10 μM). While fentanyl exposure-increased fEPSPs amplitude was prevented by picrotoxin [an inhibitor of γ-aminobutyric acid receptor (GABAR)] treatment or fentanyl washout, pretreatment of picrotoxin failed to prevent the fentanyl-impaired long-term potentiation (LTP) of synaptic strength as well as the fentanyl-enhanced long-term depression (LTD). These results demonstrated that fentanyl acute exposure and washout increases hippocampal excitability in the Schaffer-collateral CA1 pathway, depending on disinhibiting interneurons after MOR activation. In addition, fentanyl acute exposure and washout modulated synaptic plasticity, but the inhibitory activation was not critical. Elucidating the detailed mechanisms for synaptic dysfunction after fentanyl exposure and washout may provide insights into POCD generation after fentanyl anesthesia.

## Introduction

Naturally produced and synthesized opioid receptor agonists are often used as anesthetics and analgesics in clinical settings. Some patients who received regional or general anesthesia for surgery experience a short-term decline after surgery in cognitive function such as learning and memory; this is known as postoperative cognitive disorder (POCD; Moller et al., 1998; Rasmussen, 2006; Newman et al., 2007). Increasing evidence suggests that aging, duration of anesthesia, and postoperative infections rather than cerebral hypoxia are associated with POCD (Moller et al., 1998; Rasmussen, 2006; Newfield, 2009; Stenvall et al., 2012). However, the mechanisms that underlie POCD development remain largely unknown.

The hippocampus is a brain structure critical for learning and memory. Long-term potentiation (LTP) and long-term depression (LTD) of synaptic strength at the glutamatergic synapses in the hippocampus are well-characterized forms of synaptic plasticity and have long been believed to be a cellular mechanism underlying learning and memory (Bliss and Collingridge, 1993; Malenka and Nicoll, 1999). In rats, electrophysiological, immunohistochemical, and *in situ* hybridization experiments have demonstrated that the hippocampus expresses a high density of opioid receptors (Zieglgänsberger et al., 1979; Lupica et al., 1992; Drake and Milner, 2002; Stumm et al., 2004; Drake et al., 2007). Along with these studies, μ-opioid receptors (MOR) are selectively expressed on GABAergic interneurons within the CA1 region of the hippocampus (Drake and Milner, 1999; Stumm et al., 2004). Because of this interneuron expression profile of MOR, acute activation of MOR in the hippocampal CA1 area increases glutamatergic excitatory neuronal transmission by hyperpolarizing interneurons and inhibiting GABA release (Capogna et al., 1993; Svoboda and Lupica, 1998). Therefore, the enhanced excitatory neuronal transmission in the hippocampal CA1 region after opioid exposure is considered a secondary effect due to its disinhibiting effect. While activation of opioid receptors in the hippocampus by acute and chronic opioid exposure have demonstrated modulation of hippocampal plasticity and memory (Yang et al., 2004; Jafari-Sabet and Jannat-Dastjerdi, 2009), the role of acute MOR activation and washout in hippocampal synaptic plasticity of the Schaffer-collateral CA1 pathway remains elusive.

Fentanyl, an agonist of MOR largely used in human patients for anesthesia and analgesia associated with surgery, has been demonstrated to decrease GABA-mediated synaptic transmission from interneurons of the CA1 region 24 h after a single dose *in vivo* treatment (Kouvaras et al., 2008), which led to an increased susceptibility of pyramidal neurons to presynaptic stimulation. In *in vitro* cultured hippocampal neurons, synaptic α-amino-3-hydroxy-5-methyl-4-isoxazolepropionic acid glutamate receptors (AMPAR) and dendritic spins were reported to be changed within 24 h of fentanyl exposure (Lin et al., 2009). In the peripheral C-fiber synapses, acute fentanyl exposure decreased presynaptic neurotransmission, but persistently potentiated neurotransmitter release from presynaptic sites when withdrawn from the acute treatment (Heinl et al., 2011). However, whether fentanyl acute exposure and washout directly affects glutamatergic synaptic function in the Schaffer-collateral CA1 pathway remains unknown.

In the present study, we exposed hippocampal slices to fentanyl and recorded its effects on glutamatergic synaptic transmission and plasticity in the presence or blockade of GABAergic inhibition. We aimed to see if fentanyl, similar to morphine, can modulate glutamatergic synaptic plasticity in the Schaffer-collateral pathway, and particularly to examine whether inhibitory neurotransmission is involved.

## Materials and methods

### Animals

This study was approved by the Animal Care and Use Committee of No. 324 Hospital of the People's Liberation Army in accordance with the International Guiding Principles for Animals Research as stipulated by the Council for International Organizations of Medical Sciences (1985). Sprague Dawley (SD) male adults (8–10 weeks) were group-housed, with *ad libitum* access to water and food in the established animal houses, a 12 h light/dark cycle and a thermoregulated environment. All efforts were made to minimize the number of rats used and their suffering.

### Electrophysiological studies

Electrophysiological recordings were performed on coronal hippocampal slices (400 μm in thickness), as described previously (Wang and Belousov, 2011). Hippocampal slices were recovered at 37°C for at least 1 h and then maintained in an interface chamber at 29°C and perfused with ACSF (124 mM NaCl, 4.4 mM KCl, 1 mM Na_2_HPO_4_, 25 mM NaHCO_3_, 2 mM CaCl_2_, 2 mM MgSO_4_, and 10 mM glucose) continuously bubbled with 95% O_2_ and 5% CO_2_. fEPSPs were recorded from the CA1 region of the hippocampus by placing both the stimulating and the recording electrodes in the CA1 stratum radiatum. Basal synaptic transmission was assayed by plotting the stimulus voltage (V) against amplitudes of fEPSP to generate input-output relations. A baseline recording was established using low-frequency stimulation (0.033 Hz; 0.1 ms impulse duration) with adjusted intensity that induced fEPSPs with ~40% of the maximal fEPSP amplitude. LTP was induced using theta-burst stimulation (10 epochs was delivered at 5 Hz and each epoch consists 4 pulses at 100 Hz). LTD was induced using 3 Hz of 900 pulses stimulation. In experiments using inhibitors, drugs were continuously perfused over slices starting at least 10 min before fentanyl exposure, unless otherwise stated. Values of fEPSP amplitude were expressed as mean ± SEM percentage change relative to their mean baseline amplitude.

### Drug treatments

Drugs were prepared as stock solutions and diluted to the final concentration immediately before use. Final concentrations and sources of the drugs were as follows: fentanyl citrate salt (0.01, 0.1, 1 μM; Sigma), picrotoxin (50 μM; Sigma), and D-Phe-Cys-Tyr-D-Trp-Orn-Thr-Phe-Thr-NH2 (CTOP, 10 μM; Sigma). Saline or dimethyl sulfoxide (DMSO) was used as vehicle control and the final concentration of DMSO control was less than 0.5% in all experiments. Incubation of hippocampal slices with drugs was performed in either a recovery chamber or interface-recording chamber as needed.

### Statistical analysis

All data are presented as mean ± standard error of the mean (SEM). One-way ANOVA followed by Fisher's *post-hoc* comparisons was used for analyzing the difference between groups, using Stat View 5.0.1 software (SAS Institute, Cary NC). *P* < 0.05 was considered significant.

## Results

### Fentanyl dose-dependently increased excitatory synaptic transmission in the CA1 region in a MOR- and GABAR-dependent manner

**Figure 1:**
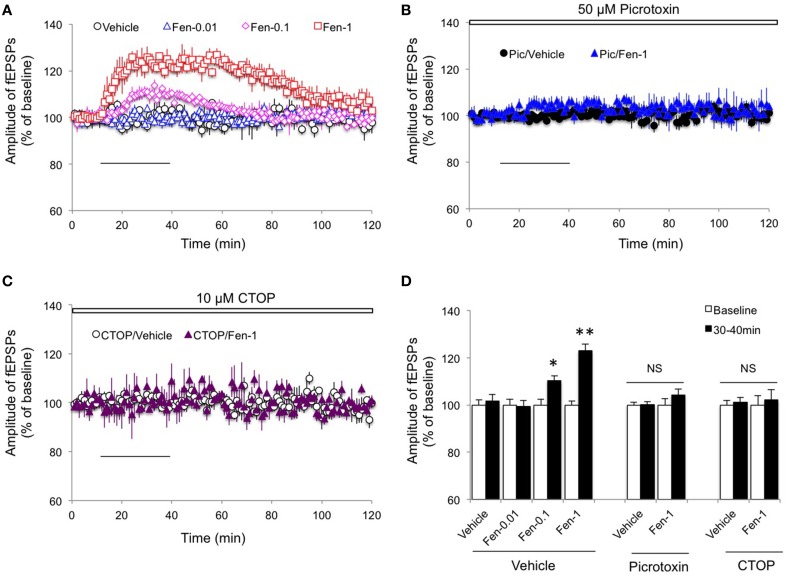
**Effect of fentanyl on basic synaptic transmission in the hippocampal CA1 area**. **(A)** The amplitude of fEPSPs was increased by fentanyl exposure (30 min, solid horizontal bar) in a dose-dependent manner. However, the elevated fEPSPs response was completely restored after a 60 min washout. Fen-0.01: 0.01 μM fentanyl; Fen-0.1: 0.1 μM fentanyl; Fen-1: 1 μM fentanyl. **(B)** In the slices with pretreatment of 50 μM picrotoxin (Pic, open bar, for at least 30 min), fentanyl exposure and washout did not change basic synaptic transmission. **(C)** In the presence of 10 μM CTOP (open bar), fentanyl exposure and washout did not change basic synaptic transmission. **(D)** Basic synaptic transmission among the indicated groups was calculated by an average of fEPSP amplitudes during baseline (0–10 min) and the last 10 min of fentanyl exposure (30–40 min). ^*^p < 0.05, ^**^p < 0.01 baseline vs. 30–40 min. Data presented as mean ± SEM. *N* = 8–12 per group from five rats.

Given that acute *in vivo* fentanyl injection decreased GABA-mediated neurotransmission from hippocampal CA1 interneurons 24 h after treatment (Kouvaras et al., 2008), we first examined if acute exposure of fentanyl to hippocampal slices increased fEPSPs amplitude in the Schaffer-collateral pathway where both presynaptic glutamate and GABA releases were integrated. As shown in Figure 1A, in the absence of GABAergic neurotransmission, lower concentration of fentanyl acute exposure did not significantly alter synaptic transmission (Figures 1A,D; *p* > 0.05 Fen-0.01 group vs. Vehicle group), but the higher concentration of fentanyl treatment significantly potentiated fEPSPs amplitudes (Figures 1A,D; *p* < 0.01 Fen-1 group vs. Vehicle group). In contrast to its role in inducing “withdrawal LTP” in C-fiber synapses (Heinl et al., 2011), the fEPSPs of Schaffer-collateral synapses were not further potentiated by fentanyl washout immediately following the acute fentanyl exposure (Figure 1A). Indeed, fentanyl-increased fEPSPs amplitude was declined to baseline levels by a 60 min drug washout (Figure 1A). Next, we evaluated the effect of fentanyl exposure and washout on excitatory synaptic transmission in the CA1 region when the GABA-mediated inhibition by interneurons was prevented. When fentanyl (1 μM) was exposed to picrotoxin (50 μM) pre-treated slices (for at least 30 min pre-incubation), there was no significant further facilitation of fEPSPs amplitude in the Schaffer-collateral pathway (Figures 1B,D; *p* > 0.05 Pic/Fen-1 group vs. Pic/Vehicle group), and fentanyl washout did not change fEPSPs either. These results suggested that fentanyl exposure indirectly promoted excitatory neurotransmission in the CA1 via inhibiting interneuron GABAergic input.

To investigate the role of MOR activation in the fentanyl-mediated increase of fEPSPs amplitude, we pretreated with CTOP (10 μM, a selective antagonist of MOR), 10 min before fentanyl exposure and washout. As we show in Figure 1C, no significant alteration in amplitude of the fEPSPs was detected (Figures 1C,D; *p* > 0.05 CTOP/Fen-1 group vs. CTOP/Vehicle group). This result indicated that activation of MOR is required for fentanyl-induced basic synaptic transmission changes.

### Fentanyl acute exposure and washout impaired LTP but facilitated LTD in the CA1 region in a inhibitory neurotransmission-independent manner

**Figure 2:**
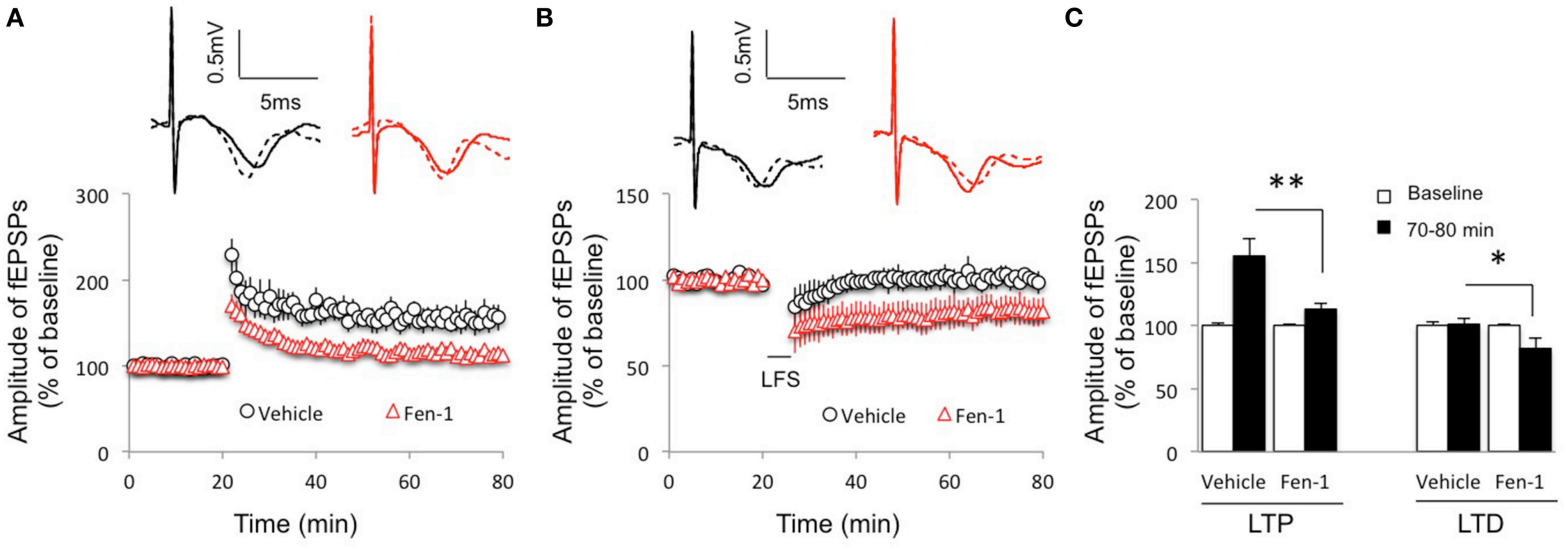
**Effect of fentanyl exposure and washout on LTP in the hippocampal CA1 area**. **(A)** Hippocampal LTP was recorded after 30 min fentanyl (1 μM) exposure and 60 min washout in the indicated groups. LTP was reduced in fentanyl-treated slices. **(B)** Hippocampal LTD was recorded after 30 min fentanyl (1 μM) exposure and 60 min washout in the indicated groups. LTD was facilitated in fentanyl-treated slices. **(C)** LTP and LTD readings among indicated groups were calculated by an average of fEPSP amplitudes during baseline (0–20 min) and the last 10 min of recordings (70–80 min). ^*^p < 0.05, ^**^p < 0.01 Vehicle-treated group vs. fentanyl-treated group. Data presented as mean ± SEM. *N* = 9–14 per group from six rats. Inserts show representative traces of fEPSP in slices treated with vehicle (black) or 1 μM fentanyl (red) before tetanus stimulation (solid line) and at the end of 1 h recording (dash line); scale bars represent 5 ms and 0.5 mV, respectively.

Since hippocampal plasticity is considered a cellular mechanism for learning and memory (Bliss and Collingridge, 1993; Malenka and Nicoll, 1999), we then evaluated the effect of fentanyl acute exposure and washout on hippocampal plasticity in the CA1 region under an *in vitro* condition. We delivered stimulations to induce LTP and LTD 1.5 h after fentanyl washout that followed a 30 min acute exposure period. The results revealed that the LTP induction was impaired by fentanyl exposure and washout (Figures 2A,C; *p* < 0.05 Fen-1 group vs. Vehicle group), with facilitation in LTD expression (Figures 2B,C; *p* < 0.05 Fen-1 group vs. Vehicle group).

**Figure 3:**
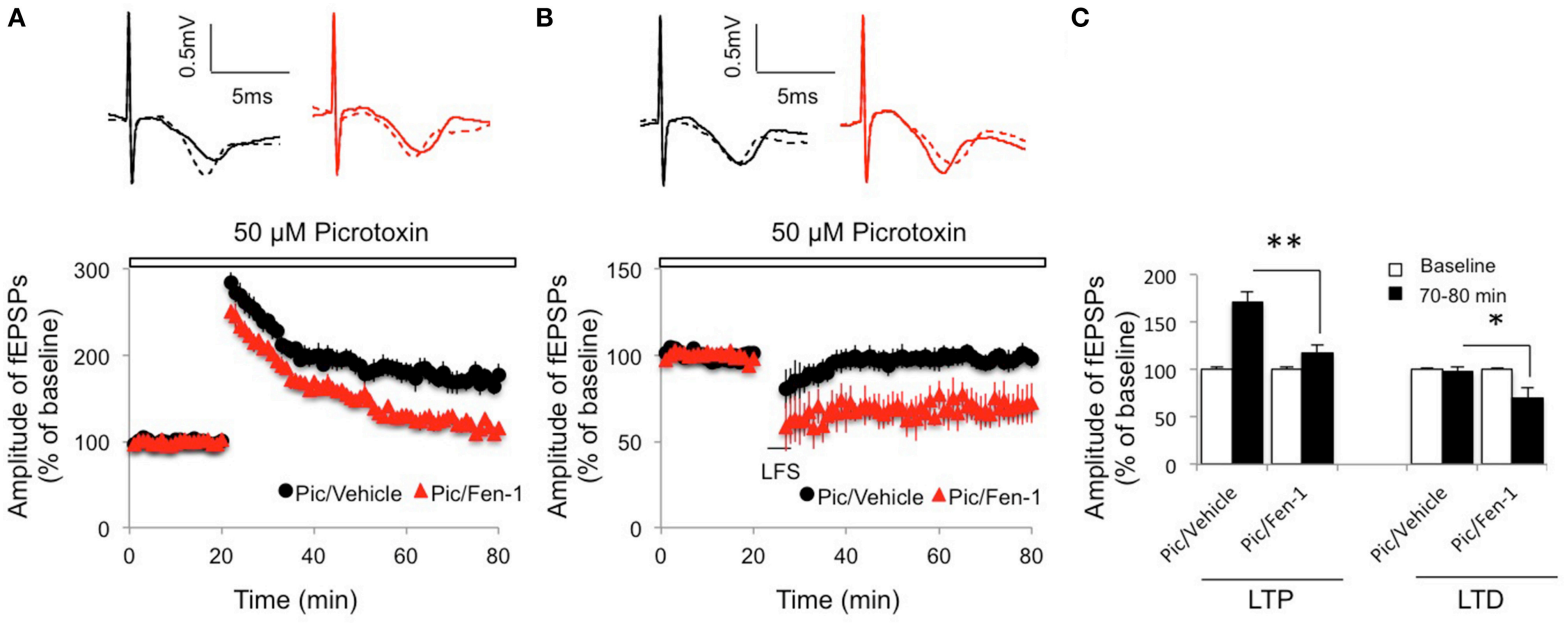
**Effect of fentanyl exposure and washout on LTP in the hippocampal CA1 area in the presence of 50 μM picrotoxin (Pic, open bar, 10 min before fentanyl/vehicle treatment)**. **(A)** Hippocampal LTP was recorded after 30 min fentanyl (1 μM) exposure and 60 min washout in the indicated groups. LTP was reduced in fentanyl-treated slices. **(B)** Hippocampal LTD was recorded after 30 min fentanyl (1 μM) exposure and 60 min washout in the indicated groups. LTD was facilitated in fentanyl-treated slices. **(C)** LTP and LTD readings among indicated groups were calculated by an average of fEPSP amplitudes during baseline (0–20 min) and the last 10 min of recordings (70–80 min). ^*^p < 0.05, ^**^p < 0.01 Pic/Vehicle group vs. Pic/Fen-1 group. Data presented as mean ± SEM. *N* = 8–12 per group from six rats. Inserts show representative traces of fEPSP in slices treated with picrotoxin/vehicle (black) or picrotoxin/1 μM fentanyl (red) before tetanus stimulation (solid line) and at the end of 1 h recording (dash line); scale bars represent 5 ms and 0.5 mV, respectively.

Given that interneurons mediated the effect of fentanyl on basic synaptic transmission (Figure 1), we next investigated if inhibitory neurotransmission was required for the modulation of fentanyl on synaptic plasticity. While LTP was slightly increased from non-fentanyl-treated hippocampal slices in the presence of picrotoxin (50 μM), a reduced LTP was observed after fentanyl exposure and washout (Figures 3A,C; *p* < 0.05 Pic/Fen-1 group vs. Pic/Vehicle group). Similarly, blockade of inhibitory neurotransmission by picrotoxin treatment did not prevent fentanyl-facilitated LTD in the CA1 area (Figures 3B,C; *p* < 0.05 Pic/Fen-1 group vs. Pic/Vehicle group).

**Figure 4:**
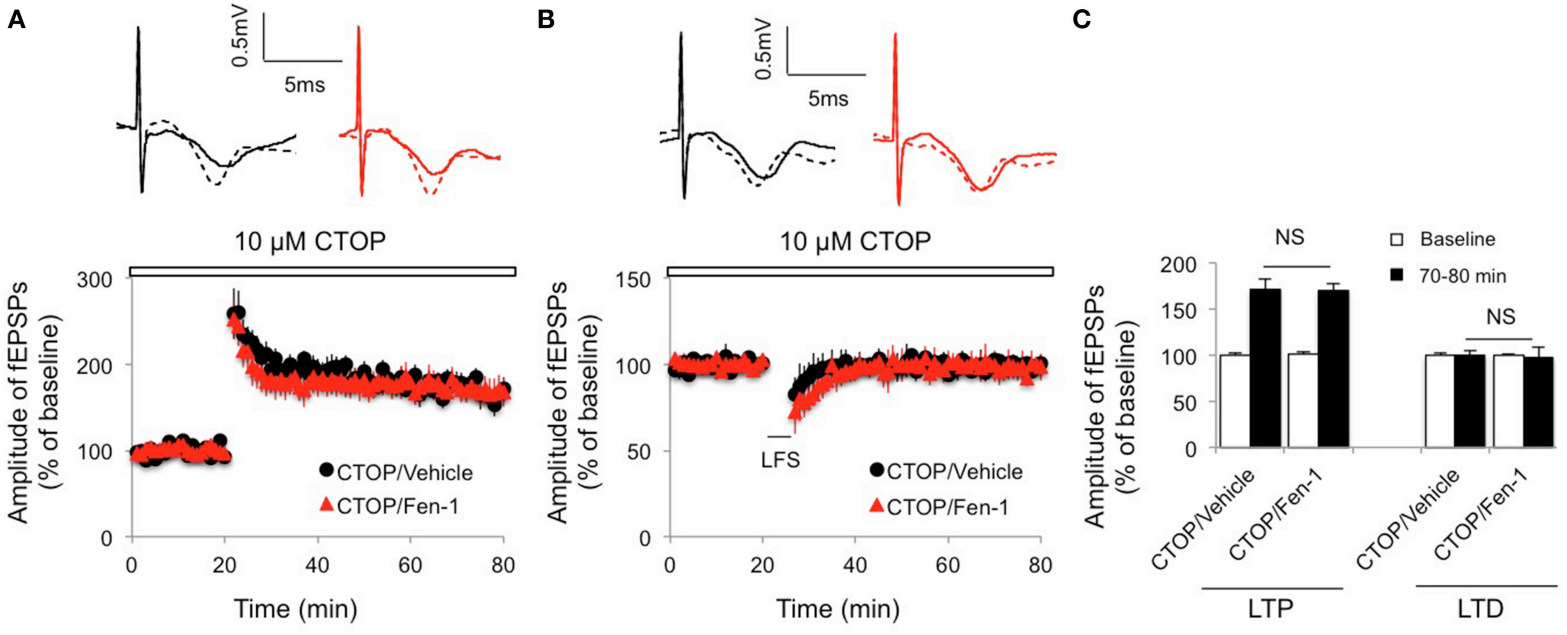
**Effect of fentanyl exposure and washout on LTP in the hippocampal CA1 area in the presence of 10 μM CTOP (open bar, 10 min before fentanyl/vehicle treatment)**. **(A)** Hippocampal LTP was recorded after 30 min fentanyl (1 μM) exposure and 60 min washout in the indicated groups. **(B)** Hippocampal LTD was recorded after 30 min fentanyl (1 μM) exposure and 60 min washout in the indicated groups. **(C)** LTP and LTD among indicated groups were calculated by an average of fEPSP amplitudes and comparisons were made between baseline (0–20 min) and the last 10 min of recordings (70–80 min). NS, non-significance CTOP/Vehicle vs. CTOP/Fen-1 group. Data presented as mean ± SEM. *N* = 8–10 per group from six rats. Inserts show representative traces of fEPSP in slices treated with CTOP/vehicle (black) or CTOP/1 μM fentanyl (red) before tetanus stimulation (solid line) and at the end of 1 h recording (dash line); scale bars represent 5 ms and 0.5 mV, respectively.

To determine if MOR is involved in fentanyl-modulated synaptic plasticity, the hippocampal slices were pretreated with CTOP (10 μM) 10 min before fentanyl exposure and the LTP and LTD inductions were recorded. We found that inactivation of MOR by CTOP completely removed the effects of fentanyl on both LTP (Figures 4A,C; *p* > 0.05 CTOP/Fen-1 group vs. CTOP/Vehicle group) and LTD (Figures 4B,C; *p* > 0.05 CTOP/Fen-1 group vs. CTOP/Vehicle group) of synaptic plasticity. These results suggested that fentanyl-modulated synaptic plasticity was the consequence of its action on MOR.

## Discussion

Many opioids such as morphine modulate basic synaptic transmission and plasticity in the hippocampal CA1 region, via activating several types of opioid receptor (μ-, σ-, κ-opioid receptors; Velísek et al., 2000; Yang et al., 2003, 2004; Dong et al., 2007, 2010; Nugent and Kauer, 2008; Hosseinmardi et al., 2009; Dacher and Nugent, 2011). In the present study, we report here that selective activation of MOR by *in vitro* fentanyl acute exposure and washout to hippocampal slices increased basic synaptic transmission in the Schaffer-collateral pathway, through its effect on disinhibition of GABAergic interneurons. More interestingly, fentanyl treatment shifted long-term plasticity of hippocampal synapses in favor of LTD induction, and the effect of fentanyl on synaptic plasticity was different from its effect on basic synaptic transmission, although both required MOR activation.

In the central and peripheral nervous systems, acute exposure to opioids activated G-protein coupled receptors, promoted potassium efflux, and inhibited calcium influx, which has been shown to generally inhibit neurotransmitter release and result in neuronal activity (Al-Hasani and Bruchas, 2011). However, opioid receptors in the hippocampus are mainly expressed in interneurons (Drake and Milner, 1999; Stumm et al., 2004), and activation of opioid receptors in interneurons reduces GABA release that normally carries inhibitory input to pyramidal neurons. Therefore, in the hippocampus, opioid exposure leads to an increased glutamatergic excitatory neuronal transmission by hyperpolarizing interneurons and GABA release inhibition (Capogna et al., 1993; Svoboda and Lupica, 1998). We should note that the effect of *in vitro* acute exposure of hippocampal tissue to opioids on CA1 synaptic transmission remains controversial. An earlier study demonstrated that acute *in vitro* application of opioid receptor agonists enhanced synaptic transmission in the CA1 area of the hippocampus (Dingledine, 1981); recently, MOR activation by opioids was also reported to increase hippocampal network activity recorded in the CA1 region (Giannopoulos and Papatheodoropoulos, 2013). However, it is also characterized by lack of effect on glutamatergic synaptic transmission by acute *in vitro* exposure of organotypic rat hippocampal slice cultures to MOR-preferring agonist D-Ala2, MePhe4, Met(O)5-ol-enkephalin (FK 33-824) in the CA3 region of the hippocampus (Capogna et al., 1993). In the present study, our results indicated that acute fentanyl exposure to hippocampal slices increased the amplitude of fEPSPs in Schaffer-collateral CA1 synapses, and this effect was prevented by inactivation of MOR or removal of GABA-mediated inhibition. Therefore, our findings support that *in vitro* acute opioid exposure can lead to an increase in CA1 basic synaptic transmission, which is indirect from the disinhibition mediated by GABA-containing interneurons. In addition, we observed that the fentanyl-increased fEPSPs *in vitro* were decreased to baseline levels within 1 h after drug washout, which was also different from the *in vivo* acute treatment in which the facilitation lasted for several hours (Yang et al., 2004). This difference might derive from the activation of MOR in other brain regions following *in vivo* injection, rather than limited to hippocampus in *in vitro* treatment.

While the *in vitro* acute effect of fentanyl on hippocampal synaptic plasticity has not been fully investigated, it is known that *in vivo* acute opioid administration facilitated hippocampal LTD in the Schaffer-collateral CA1 pathway (Yang et al., 2004). In addition, opioid exposure during the prenatal period was reported to impair hippocampal LTP and facilitate LTD induction in Schaffer-collateral synapses (Velísek et al., 2000; Yang et al., 2003). Similar to acute *in vivo* administration, chronic exposure to opioids has been shown to reduce LTP in the CA1 area of the hippocampus in both *in vivo* (Pu et al., 2002) and *in vitro* experiments (Salmanzadeh et al., 2003). Our results in the present study revealed that acute fentanyl exposure and washout suppressed hippocampal LTP but facilitated LTD expression in the Schaffer-collateral pathway.

The significant finding of the present study was that activation of MOR by fentanyl might target distinct mechanisms for modulating basic synaptic transmission and activity-dependent synaptic plasticity. For the increase of basic excitatory synaptic transmission, the fentanyl-induced MOR activation in interneurons caused disinhibition of excitatory synaptic connection and transmission, which has been well described in the literature (Al-Hasani and Bruchas, 2011). However, our findings of the effect of fentanyl on synaptic plasticity seemed independent from GABA-mediated inhibitory neurotransmission within the CA1 region, despite the fact that activation of MOR was still required. In addition to the MOR-activation coupled inhibitory neurotransmission, newer findings have suggested that MOR activation also modulated other MAPK signal transduction pathways, including P38 (Tan et al., 2009), JNK (Macey et al., 2006), and ERK (Tan et al., 2009). Therefore, the alterations of these signaling transduction pathways following the MOR activation by fentanyl application might play a critical role in activity-dependent synaptic plasticity. In addition, activation of MOR in astrocytes has been reported (Miyatake et al., 2009). In the bath application of fentanyl in our present study, MOR activation might have also induced MAPK signaling transduction change among astrocytes, which could affect local environment stress and inflammation that eventually shaped the synaptic plasticity on the excitatory neurotransmission connections.

In addition, the bath application of fentanyl would globally activate MOR, including the interneurons within the CA1 region. Given that MORs are expressed in interneurons of other areas of the hippocampus such as in the CA3 region and dentate gyrus, the *in vitro* acute fentanyl exposure and washout to hippocampal slices could also activated MOR in those regions, which may affect Schaffer-collateral excitatory synaptic plasticity. Although our present study did not dissect a detailed pathway, it might provide a starting point for further investigations into the underlying mechanism of synaptic plasticity modulation induced by acute exposure and washout of MOR agonists.

Together our results indicate that the basic synaptic transmission in Schaffer-collateral CA1 synapses was increased by *in vitro* acute fentanyl exposure and washout, which is secondary to MOR activation-mediated GABA disinhibition among interneurons. However, *in vitro* acute fentanyl exposure and washout also shifted synaptic plasticity in favor of synaptic depression using a different mechanism. Understanding the underlying mechanism of fentanyl-mediated synaptic plasticity alterations might provide some clues for cognitive impairment after clinical fentanyl use in patients.

## Author contributions

HT designed and performed experiments. YX and FL performed experiments and statistical analysis. GW arranged and supervised the drug purchasing and usage. HT and SH wrote the paper.

### Conflict of interest statement

The authors declare that the research was conducted in the absence of any commercial or financial relationships that could be construed as a potential conflict of interest.
